# A retrospective cohort study on the cost-effectiveness analysis of kidney transplantation compared to dialysis in Cameroon: evidence for policy

**DOI:** 10.11604/pamj.2023.46.27.38706

**Published:** 2023-09-18

**Authors:** Rene Kanjo Njamnshi, Mahamat Maimouna, Leonard Ngarka, Aristide Eric Nono Tomta, Wepnyu Yembe Njamnshi, Gloria Enow Ashuntantang, Vincent de Paul Nguemaleu Djientcheu, Alfred Kongnyu Njamnshi, Donald Sloane Shepard

**Affiliations:** 1 Heller School for Social Policy and Management, Brandeis University, Waltham, Massachusetts, United States of America,; 2 Brain Research Africa Initiative (BRAIN), Yaoundé, Cameroon,; 3 Haemodialysis Unit, Yaoundé General Hospital, Yaoundé, Cameroon,; 4 Department of Internal Medicine and Specialties, Faculty of Medicine and Biomedical Sciences, The University of Yaoundé I, Yaoundé, Cameroon,; 5 Neuroscience Laboratory, Faculty of Medicine and Biomedical Sciences, The University of Yaoundé I, Yaoundé, Cameroon,; 6 Department of Clinical and Developmental Neuropsychology, University of Bournemouth, England, United Kingdom,; 7 Clinical Neuroscience Division (Neurology-Neurosurgery), Yaoundé General Hospital, Yaoundé, Cameroon,; 8 Department of Surgery and Specialties (Neurosurgery), Faculty of Medicine and Biomedical Sciences, The University of Yaoundé I, Yaoundé, Cameroon

**Keywords:** Kidney transplantation, haemodialysis, Cameroon, retrospective studies, chronic kidney failure

## Abstract

**Introduction:**

chronic kidney disease affects one in ten adults in Cameroon. Haemodialysis was the only renal replacement therapy (for adults) in Cameroon and its sub-region until November 10, 2021. Thereafter through May 2022, the Yaoundé General Hospital successfully completed four living-donor kidney transplants. This paper examines policy implications.

**Methods:**

medical records of cohorts of kidney failure patients who started haemodialysis at Yaoundé General Hospital in 2012 (n=106) and 2017 (n=118) were abstracted retrospectively through 2021 and their survival analyzed with Microsoft Excel and Kaplan-Meier curves. Using hospital data, the literature, and price indexes, the annual medical cost per patient of dialysis and living-donor kidney transplantation in 2022 prices was derived.

**Results:**

the 9.5-year survival rate for the 2012 cohort was 11% and the 5-year rate for the 2017 cohort was 18%. Annual haemodialysis cost per patient averaged $17,681 (26.5% from households and 73.5% from government). Initial transplantation costs averaged $10,530 per patient, all borne by the government. Under the brand-drug option, first-year transplantation follow-up costs $19,070 (4% for laboratory and 96% for drugs).

**Conclusion:**

annually, haemodialysis in Cameroon costs per patient 12 times the country's average income ($1,537), driven especially by the costs of equipment purchase, maintenance, and consumables. Cameroon's initial cost of transplantation is lower than in other African countries. Generic drugs could lower annual follow-up costs by 89%. If Cameroon could achieve long-term survival with generic drugs after kidney transplantation, that modality would become a reasonable option for selected kidney failure patients (e.g. younger and without other comorbidities).

## Introduction

Chronic kidney disease (CKD) affects one in ten Cameroonian adults, with a higher prevalence in rural than urban areas, and imposes substantial morbidity, mortality and costs to government and families [1]. One Cameroonian study reported that survival on haemodialysis averaged only 8 months and most patients died within the first 3 months [2]. Another study reported more than a quarter of haemodialysis patients dying within the first year of starting dialysis, with most deaths occurring within the first six months [3]. Although Cameroon lacks a dialysis registry, experts estimated that Cameroon had about 1,500 patients on haemodialysis across 14 centers (12 public and 2 private) as of December 31, 2021. These centers are spread across all 10 regional capitals including two in the political capital (Yaoundé), and two in the commercial capital and largest city (Douala). Four (28.6%) of the 14 centers (Yaoundé General Hospital (YGH), Douala General Hospital, Garoua General Hospital, and Yaoundé University Teaching Hospital (CHU)) are all first (highest) category hospitals. These highest category hospitals receive legal and financial autonomy.

In Cameroon, most end-stage kidney disease (ESKD) patients receive two weekly 4-hour haemodialysis sessions. In addition, one health facility (Mbingo Baptist Hospital in the North West Region) performs peritoneal dialysis, specifically for children with acute kidney injury.

A previous cost analysis of 155 patients in three public-sector facilities in Cameroon concluded that maintenance haemodialysis cost $13,581 (7,988,761 Central African CFA Franc, XAF) per year per patient, *excluding staff and building costs* in 2012-13 [4]. The included costs comprised direct medical costs, mainly dialysis consumables (87.7%), transportation (5.7%) and productivity loss for patients and their caretakers during dialysis sessions (6.6%). Although Cameroon's national government subsidizes haemodialysis, the out-of-pocket costs are $4,114 (2,420,255 XAF), creating a heavy burden for dialysis patients and their families. The out-of-pocket costs represent 2.68 times Cameroon´s Gross Domestic Product (GDP) per capita of $1,537 [5], 5.56 times Cameroon´s minimum wage of $740/year (36,270 XAF/month) [6], 5.93 times the World Bank´s extreme poverty line for 2017-2021 ($1.90/day or $694/year) [7], and 5.24 times the World Bank´s 2022 extreme poverty line ($2.15/day or $785/year) [8]. As the World Health Organization considers health expenditures exceeding 25% of household income as catastrophic [9], haemodialysis dialysis constitutes a catastrophic health expenditure for most Cameroonians.

Dialysis also poses a substantial challenge for the government. Public hospitals have insufficient working machines and other resources to satisfy need, forcing nephrologists to make difficult rationing choices in the absence of national allocation guidance [10-12]. Previous studies on haemodialysis costs in Cameroon [4] excluded fixed and semi-variable costs, and none assessed the cost-effectiveness of haemodialysis or transplantation in Cameroon. On November 10, 2021 Cameroon performed its first kidney transplant, followed by three more in May 2022. To inform policy, this study derives and compares the costs and outcomes of haemodialysis and kidney transplantation in Cameroon.

This study has three objectives. First, we seek to determine survival on dialysis for two cohorts of patients diagnosed with CKD. Second, we seek to increase knowledge on cost of dialysis and kidney transplantation in Cameroon to allow for more accurate measurement and cost-effectiveness of kidney transplantation in Cameroon compared to haemodialysis. Third, we seek to conduct a cost-effectiveness analysis of transplantation compared to haemodialysis for KF. We hypothesize that kidney transplantation economically dominates haemodialysis being superior in projected health outcomes and lower projected lifetime costs.

## Methods

**Study design:** this is a retrospective cohort study of two cohorts of haemodialysis patients and one cohort of kidney transplant recipients, all from YGH. The study was designed to inform policy on the choice between haemodialysis and kidney transplantation in patients with ESKD. The study first extracted and analyzed data from records of ESKD patients who were on maintenance haemodialysis during the study periods. The study then determined the cost of haemodialysis and kidney transplantation. Medical data were extracted from patients´ records by qualified medical personnel. No patients were contacted for the study and no personal data were disclosed. The YGH general manager authorized and gave ethical approval for extraction and analysis of data from existing medical and administrative records to inform treatment.

**Setting:** this study was carried out in YGH in Cameroon. Data were extracted from January 1 through March 31, 2022 from patients´ medical records and death data. Yaoundé, the capital of Cameroon and the center region headquarters, has two hemodialysis centers - one at the YGH and the other at the University Teaching Hospital. At the end of 2021, Cameroon had 28 nephrologists compared to 5 in 2012 [3]. The YGH haemodialysis unit had 16 functional dialysis machines (11 Fresenius and 5 Nikkiso, all bought between 2016 and 2021) and 23 medical staff (4 nephrologists, 1 major who is a nurse, 2 nursing assistants and 16 nurses) during the study period. The hemodialysis unit at the YGH has capacity to have 25 hemodialysis machines. The unit runs 24/7 and handles the bulk of the country´s dialysis patients. As in other dialysis centers in Cameroon, patients are scheduled for two dialysis sessions of four hours each per week. There were 186 hemodialysis patients receiving treatment at this center as of March 2022.

**Participants in the retrospective analysis of haemodialysis survival:** the participants were two retrospective cohorts of patients with ESKD initiating long-term hemodialysis at the haemodialysis unit of the YGH in Cameroon. Participants were identified through the YGH register of ESKD patients. All patients who initiated maintenance haemodialysis at the YGH haemodialysis unit in 2012 or 2017 constituted the 2012 and 2017 cohorts, respectively. Patients who had initiated dialysis prior to those years were not part of the study cohorts.

**Participants in the retrospective analysis of kidney transplantation:** kidney transplant participants were all patients who received a transplant in Cameroon between 10 November 2021, the date of Cameroon´s first kidney transplant, through May 2022. Only short-term follow-up data were available for transplant patients.

**Variables in the retrospective analysis of haemodialysis survival:** our dependent variable is survival in years from the initiation of haemodialysis until the date of the patient´s death, or the end of follow-up if the patient was then still alive. Our independent variable is the cohort to which each patient belonged based on the year in which dialysis was first initiated, 2012 or 2017. To describe our population, age group and gender were tabulated. As comorbidities (e.g. hypertension, obesity, HIV, and diabetes mellitus) have been previously studied [1], we focused here on cost and long-term survival.

**Characteristics of haemodialysis patients:** medical records of ESKD patients who started haemodialysis at the haemodialysis unit of YGH were reviewed for each cohort. Extracted data included the patient´s age, sex, month and year dialysis was initiated, and month and year of death or loss to follow-up (if applicable). Medical data were extracted from patients´ records by physicians and other qualified medical personnel.

**Cost data:** for haemodialysis, variable costs were obtained from a November 2012 to April 2013 study [4] and categorized by payer between government and out-of-pocket. Where the original source reported a range or multiple values, we used the median. The placement of electricity and water under “non-medical” costs follows that of the source. Most other direct costs were obtained from the haemodialysis service, pharmacy unit, and billing center at YGH. Remaining items (e.g. market rental rates and costs of general furniture) were obtained from local market surveys (Annex 1). Lifespans of office equipment were obtained from Cameroon General Tax Code 2021. Semi-variable costs consist of medical staff salaries, contracts for cleaning and security, rental value of space. Staff salaries unit cost are the global monthly salary for 23 health workers. Quantities are the number of months in the year. Aggregate pay for 23 medical staff at 2,875,000 FCFA (XAF) per month. Building: cost is monthly rental rate for similar space in neighborhood. As at March 2022, the haemodialysis unit of YGH was dialyzing 186 patients. Average costs per patient of haemodialysis were thus obtained by dividing total costs by 186.

Overhead covered support services (e.g. laundry, billing, and general hospital administration). Overhead is assumed to be 15% of total direct cost based on policies of the Bill and Melinda Gates Foundation for grants in low- and middle-income countries). Expenses from the November 2012 to April 2013 study were converted at that study's current exchange rate (1 US dollar (US$) equals 588 XAF) [4]. As this exchange rate approximates subsequent values (January 1 through December 31, 2022, ranging from 550 to 635 XAF per US$), it has been used throughout this study.

Initial transplantation and post-kidney transplant follow-up cost data were obtained for Cameroon´s first four transplants from the general management of YGH, the pharmacy and billing departments. The annual cost was derived from monthly follow-up costs obtained for the months of December 2021 through March 2022. Annualized costs amortized the first-year transplant cost over the assumed 10 years duration of the transplant using the standard economic discount rate of 3% per year. Costs reported here represent the Cameroonian team, but do not include the time, transportation, and accommodation of the Swiss team. Laboratory testing and costs are based on the Cameroonian experience.

**Minimizing bias:** bias could arise if the haemodialysis and transplant recipients varied on demographic characteristics that were related to survival, or if missing data created marked differences between analytical and original cohorts. As only limited cohort data were obtained, unmeasured characteristics could explain differences among the 2012 and 2017 haemodialysis cohorts and the 2021-22 transplant cohort. For example, transplant patients might have been selected for young age and/or absence of comorbidities. Both haemodialysis cohorts had low and comparable proportions with unknown survival (5.6% (6 out of 106) from 2017 and 5.9% (7 out of 118)) from 2017, despite differences in study durations. The same team of researchers, who were not involved in either treatment modality, analyzed cost and outcomes for both transplantation and dialysis, minimizing researcher bias. Both modalities occurred in the same hospital in Cameroon. The researchers measured and acknowledged any remaining sources of bias due to the selection of transplant recipients.

**Study populations:** the haemodialysis study population consisted of the 224 patients in the 2012 and 2017 cohorts. Follow-up, based on records at YGH through December 31, 2021, recorded whether the patient was known to be at the end of follow-up, and if not, date of death or of last contact. Altogether, 211 patients had complete follow-up and the remaining 13 had partial follow-up (status and whereabouts unknown, including transfer to another center). All patients were included in the analysis with censoring for patients lost to follow-up.

Before data collection, we hypothesized 50% survival at follow-up, chosen as the midpoint of the possible range. Using the normal approximation to the binomial formula, we calculated that the 95% confidence interval for survival would be 40%-60% for the 2012 cohort, and 41% to 59% for the 2017 cohort. For transplantation, a similar analysis with only 4 patients showed the 95% confidence interval range would be 1% to 99%. That range is so wide to be not informative. Furthermore, one would need to wait 5 years to obtain data. To address policy about transplantation in Cameroon now, modeling based on literature was used instead.

**Quantitative variables:** they involved in the study include patient´s age at dialysis initiation, survival time and costs. Age groupings in our study followed the life table (life expectancy and mortality for various age groups) for Cameroon for 2019. Although the aim of this study was not to estimate the CKD burden of different age groups or dwell in details of relationship between age, CKD, and deaths, using the life table opens an opportunity to look into such details for someone interested in that aspect of the research.

Date data were collected only on the month (and not exact day) when dialysis was initiated, and when a patient died. To calculate live period for those patients who died, we considered the fifteenth (mid-point) of each of the months of event. For censored data, we considered the last day of the year prior to that when the patient was lost to follow-up, and December 31^st^ for those who were still alive at the end of the haemodialysis study period, December 31, 2021. Costs were derived in local currency and converted into United States Dollar (USD) at 1 USD equals 588 XFA for consistency with a previous costing study.

**Statistical methods:** data analyses were performed on all 224 patients. Descriptive statistics (counts, percentages, means, medians and standard deviation [SD]), amortization, sensitivity analyses, and cost-effectiveness analyses were conducted with Microsoft Excel Professional Plus 2019 (Microsoft Corporation, Redmond, WA). The Kaplan-Meier estimator was used to compute survival on dialysis by month and cohort using Stata version 16 (LLC Stata Corp., College Station, TX). Under this procedure, patients lost to follow-up were included up to the end of their last month in which their status was known. Survival of the 2012 cohort was determined not only at 9.5 years, but also at 5 years from the initiation of dialysis to facilitate comparison with the 2017 cohort.

## Results

**2012 haemodialysis cohort:** of the 106 participants in the 2012 haemodialysis cohort, 59% were men and 41% were women. Patients’ ages ranged from 9 to 77 years, with 54% aged 50 years or older and an average (± standard deviation) of 49 (±13.47) years. Follow-up time averaged 4.72 years. Six of 106 (5.6%) patients in this cohort were lost to follow-up at some point.

**2017 haemodialysis cohort:** of the 118 participants in the 2017 haemodialysis cohort, 60% were men, 40% were women, and 64.5% were 50 years or older when they began haemodialysis. Their age averaged 54.7 (±14) years. Their follow-up time averaged 2.50 years. Seven of 118 (5.9%) patients in this cohort were lost to follow-up at some point.

**2021-22 transplant cohort:** the transplant cohort consisted of four transplant recipients--two males (ages 28 and 34) and two females (ages 32 and 33). Their average age (± standard deviation) at kidney transplantation was 32 (±2.6) years. As of September 2022, 1.8 person-years of follow-up were available on the four transplant recipients. This was based on 10 months (December 2021 to September 2022) post-transplant period for the first transplant and 4 months (June to September 2022) each for the three transplant recipients in May 2022.

**Survival on haemodialysis:** age at dialysis initiation ranged from 9-77 years (median 50) in the 2012 cohort and 9-80 years (median 56) in the 2017 cohort (Table 1). The 9.5-year survival rate for the 2012 cohort was 10.37% with a 95% confidence interval of 4% to 32%. Its 5-year survival rate was 47% with a 95% confidence interval of 37% to 56%. For the 2017 cohort, the 5-year survival rate was 16.9% with a 95% confidence interval of 18% to 48%.

**Table 1:** description of cohorts at initiation of haemodialysis

	2012 cohort		2017 cohort	
Category	N	%	N	%
	**Overall distribution**			
Male	63	59%	71	60%
Female	43	41%	47	40%
Total	106	100%	118	100%
**Breakdown by age (years)**				
5-9 years	1	1%	1	1%
10-14 years	1	1%	2	2%
15-19 years	1	1%		0%
20-24 years	4	4%	1	1%
25-29 years	5	5%	4	3%
30-34 years	3	3%		0%
35-39 years	7	7%	3	3%
40-44 years	12	11%	11	9%
45-49 years	15	14%	20	17%
50-54 years	17	16%	12	10%
55-59 years	14	13%	15	13%
60-64 years	16	15%	20	17%
65-69 years	5	5%	14	12%
70-74 years	4	4%	9	8%
75-79 years	1	1%	5	4%
80-84 years			1	1%
Total	106	100%	118	100%

**Figure 1:**
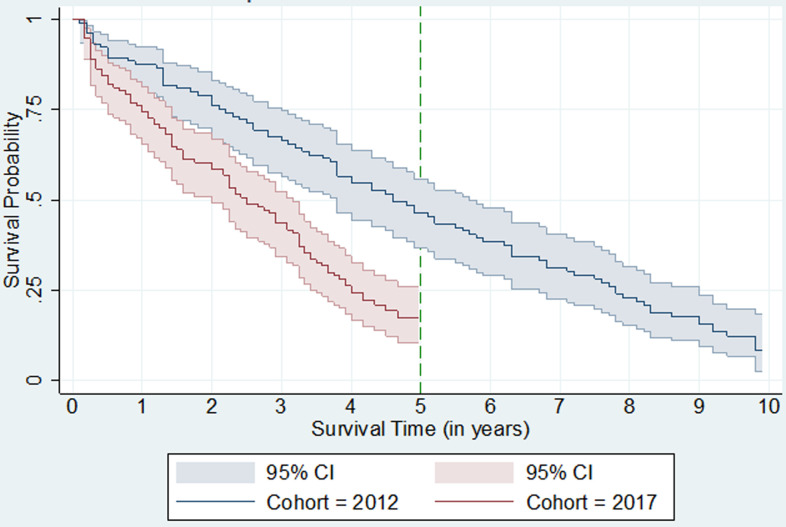
Kaplan-Meier survival estimates: 2012 and 2017 cohorts

Within the 9.5-year study period for the 2012 cohort, all patients averaged 4.8 years alive while those who are known to have died averaged 4.2 years alive. Within the 4.5-year study period for the 2017 cohort, all patients averaged 2.5 years alive while those who are clearly known to have died averaged 2.1 years alive. In the 2017 cohort, 21 (23%) of the 91 deaths occurred during the first year of haemodialysis. The median survival was 4.77 years for the 2012 cohort and 2.84 years for the 2017 cohort (Figure 1).

**Cost of haemodialysis:** annual haemodialysis maintenance costs the health system $17,681 per patient, comprised of $4,687 (26.5%) out-of-pocket and $12,994 (73.5%) costs to government (Table 2 and Annex 2). Assuming complete participation (104 annual sessions, based on 2 sessions per week times 52 weeks), the medical costs would have averaged $170 (i.e. $17,681/104) per patient per session. In addition to these reported medical costs, the average other items per patient per year were non-medical fixed costs ($9.30), transport ($208), and meals ($122).

**Table 2:** annual medical cost per patient on maintenance haemodialysis in Cameroon by payer (US$)

Item	Patient (out-of-pocket)	Hospital and government	Total	Column %
**Fixed costs**				
Fixed cost (medical)	$0	$1,566	$1,566	8.9%
Semi variable costs	$0	$558	$558	3.2%
**Direct variable costs**				
Direct variable costs (medical)	$3,785	$8,120	$11,905	
Dialysis water and electricity	$0	$444	$444	
Total direct variable costs	$3,785	$8,564	$12,349	69.8%
Indirect variable costs	$902		$902	5.1%
Total fixed and variable costs	$4,687	$10,687	$15,374	87.0%
Overhead costs: 15% of total fixed and variable costs for hospital and government		$2,306	$2,306	13.0%
Overall annual cost	$4,687	$12,994	$17,681	100.0%
Row (%)	26.5%	73.5%	100.0%	

**Cost of living donor kidney transplantation:** the four kidney transplants were performed by a joint team of Swiss and Cameroonian surgeons and other medical personnel in November 2021 and May 2022, with an initial average cost per transplant of $10,530 (Table 3). All of these costs were borne by the government. The amounts in this table do not include the cost of routine hospitalization (hotel and general nursing), which the authors estimated at an additional $552 for the donor and recipient, per transplant. Including these routine hospitalization costs, the overall initial transplant cost averaged $11,082.

**Table 3:** initial medical costs per case (excluding follow-up) of transplantation in Cameroon in 2022 US$

Item	Kidney recipient	Kidney donor	Total
	**First case**		
Pre-workup	310	167	477
Surgery	2,196	1,726	3,921
Medication	1,755	1,585	3,339
Total	4,261	3,477	7,738
	**Second case**		
Pre-workup	1,504	374	1,878
Surgery	3,207	3,070	6,277
Medication	2,093	2,171	4,265
Total	6,804	5,615	12,420
	**Third case**		
Pre-workup	618	264	881
Surgery	2,773	1,496	4,269
Medication	2,220	2,402	4,621
Total	5,610	4,161	9,771
	**Fourth case**		
Pre-workup	530	374	904
Surgery	3,717	1,549	5,266
Medication	2,938	3,082	6,020
Total	7,184	5,005	12,190
	**Average, all cases**		
Pre-workup	740	295	1,035
Surgery	2,973	1,960	4,933
Medication	2,251	2,310	4,561
Total	5,965	4,565	10,530

Under the brand drug option, first-year transplantation follow-up (excluding the transplant) costs $19,070 per transplant recipient (4% for laboratory and 96% for drugs). This includes pre-transplant work-up, surgery, medication, 10 hospital days for the recipient, and 2 hospital days for the donor. It does not include productivity loss for patients; costs for transportation, accommodations, and salaries for the Swiss team, as their primary purpose was capacity building in Cameroon. Using brand drugs brought the overall first-year cost to $30,152 ($19,070 + $11,082). Total 10-year annualized cost was $19,929 per year with brand drugs (Table 3, Table 4, Annex 3, Annex 4, Annex 5).

**Table 4:** follow-up and annualized cost of transplantation per recipient

Item	Cost (USD)
**Annual follow-up costs: brand drug option**	
Laboratory tests (routine)	$694
Brand drugs	$18,376
Overall annual follow-up cost (laboratory plus brand drugs)	$19,070
**Annual follow-up costs: generic drug option**	
Laboratory tests (routine)	$694
Generic drugs	$1,470
Overall annual follow-up cost (laboratory plus generic drugs)	$2,163
**Overall annualized costs under alternative lifespans**	
Brand drug option (10 years)	$19,929
Generic option (5 years)	$3,763
Generic option (10 years)	$3,022
Generic option (15 years)	$2,777

Alternatively, if generic drugs (e.g. cyclosporine) had been used, annual follow-up cost would have averaged $2,163 (1,272,105 XAF) per transplant recipient [13]. Thus, generic drugs would have reduced the follow-up cost by 89% compared to brand drugs (i.e. $2,163 versus $19,070). If the financing of generic follow-up costs followed the haemodialysis allocation, the government would pay $1,590 (73.5%) and the family would pay $573 (26.5%) per transplant recipient annually.

**Cost-effectiveness of alternative treatments:** based on their experience, the study's clinical authors estimate that with no treatment, median survival would have been 0.25 years and health expenditure per patient would have been Cameroon's per capita average. The authors used the median survival from the latest (2017) cohort (2.84 years) for haemodialysis and estimated roughly 10 years (considering current conditions in Cameroon's health system) for living donor transplantation. The incremental cost-effectiveness analysis found that compared to no treatment (ESKD is fatal without treatment), haemodialysis added 2.59 discounted years at an incremental cost of $19,455 per year, while transplantation added 9.75 discounted years at a cost of $3,110 per year. Transplantation economically dominates haemodialysis by adding 7.16 more expected years alive while saving $21,678 per patient in lifetime medical costs (Table 5).

**Table 5:** cost-effectiveness calculations

Item	Years alive	Cost per year alive (USD)	Discounted years alive	Present value cost (USD)	Incremental cost-effectiveness ratio (ICER)
**Treatment options**					
No treatment	0.25	$54	0.25	$13	n.a.
Haemodialysis (2017 cohort)	2.84	$17,681	2.68	$47,456	n.a.
Transplantation	10	$3,022	8.53	$25,778	n.a.
**Differences between treatment options**					
Haemodialysis vs. no treatment	2.59	n.a.	2.44	$47,443	$19,455
Transplantation vs. no treatment	9.75	n.a.	8.28	$25,765	$3,110
Transplantation vs. haemodialysis	7.16	n.a.	5.85	-$21,678	-$3,708

n.a.: not applicable; ICER: incremental cost per incremental year alive.

## Discussion

Using cohorts and modeling, this study compared costs and outcomes of haemodialysis and transplantation for patients with ESKD. This study, like previous ones [2,10], confirmed that mortality on haemodialysis in Cameroon is high. The 16.9% 5-year survival rate for the more recent cohort is low by international standards. For example the 5-year survival rates were 50% in a Brazil cohort study [14] and 35% for all hemodialysis recipients in the United States [15]. Each person-year on dialysis is a substantial expense for the government of Cameroon. Out-of-pocket costs can be catastrophic for most households [9].

Potentially modifiable reasons for these high death rates and short life expectancy on haemodialysis in Cameroon include late referrals of patients [4], breakdowns of haemodialysis machines leading to missing sessions [11,12], failure or infection of the vascular access (due to prolonged use of catheters) and inadequate anemia management. In this study, the younger age of transplant recipients is a potential confounder in comparing survival between dialysis and transplantation.

The annual cost of haemodialysis to the healthcare system is high: $17,681 per patient and $3.29 million for 186 patients. Purchase and maintenance of good quality equipment, YGH´s current approach, is expected to minimize complications and control long-run costs. Despite substantial government subsidy, out-of-pocket payments for maintenance haemodialysis are still formidable for most families given Cameroon's low GDP per capita [4]. Thus, kidney transplantation is considered the better treatment option for CKD.

Globally, a patient´s lifespan post-kidney transplantation (living donor kidney) is reportedly 15 to 20 years longer than on haemodialysis [16]. Short term transplant outcomes in Cameroon are encouraging. Following the country's first transplant (November 2021) and three more transplants (May 2022), donor and recipients remained well as of December 2022, after 11 and 7 months, respectively. The initial cost of transplantation in Cameroon ($10,530) is comparable to that in Tanzania ($10,000) and in private hospitals in India ($6,569-$7,883) [17], but lower than in other African countries ($32,000 in Nigeria, and $18,775 in Ghana) [18] and public hospitals in India ($13,000-$17,000) [19]. India's annual follow-up cost post-transplantation averaged around $2,358, including $1,572 in immunosuppressants and other drugs [17]. India's position as the world´s largest supplier of generic drugs may have contributed to its low post-transplant follow-up costs. The use of generic drugs, such as cyclosporine, can help lower follow-up costs [11], and is planned for future use in Cameroon.

More consistent implementation of clinical guidelines for screening and management of risk factors, such as hypertension and diabetes, might help prevent ESKD and its costs in the future. The use of generic drugs would make transplantation more cost-effective than dialysis and is the preferred alternative. With a recent per capita healthcare cost of $54 [20], Cameroon can afford transplantation or haemodialysis for only a small number of patients. To maximize equity and outcomes, these patients could be selected based on a more stringently applied medical score of predicted post-transplant survival. Adapting international criteria, this score could use recipient and donor-related characteristics, including young age, few or no co-morbidities, and first-degree or other close relationship between donor and recipient [21]. The program may wish to examine whether limiting the number of patients to ensure spare machine capacity and robust financing might result in greater aggregate survival.

Although initial transplant costs of all four recipients were covered by the government, such financing would not be possible for every patient needing a transplant given the government´s limited resources. Hence, family contributions may be needed. Because brand drugs are unaffordable to almost all Cameroonian families and prescribing them would likely cause poor adherence, prescribing generic drugs will likely lead to better survival of the graft and the transplant recipient. As a lower-middle income country, the public resources that Cameroon can allocate to CKD are necessarily limited. The annual healthcare cost of a haemodialysis patient ($17,681) is 327 times Cameroon´s per capita health care costs. Although annualized health care costs with generic drugs are much lower ($3,022), they are still 56 times the country´s per capita health expenditures.

A casual response to public resource constraints might be to require a much larger family share (e.g. 50%) of annualized medical costs. These would be $8,841 for hemodialysis and $1,511 for transplantation. To ensure that a family could afford such expenses without their becoming catastrophic [9], a participating family would need an income of 48 and 8 times the minimum wage, respectively. Thus, only very high-income families would have sufficient incomes to support these out-of-pocket expenses. As the government would still be funding the other half of costs, this casual response would be highly regressive, directing government funding to the highest income families.

To promote equity, a different type of approach would be needed. For example, a family´s contribution might be assessed on a sliding scale. To operationalize this idea, the family's required share of costs might be based on socio-economic categories such as Rwanda´s Ubudehe system [22], thereby directing government subsidy to the poorest patients. Lessons from South Africa´s ‘Accountability for Reasonableness´ approach [23] may identify future refinements.

Several limitations should be acknowledged. The transplantation program has data for only the first four patients and ten months of follow-up for this paper. Survival could be lowered substantially by transplant rejection or infection due to suppressed immunity. However, our sensitivity analysis (Table 4, Annex 5) shows that even if survival were as short as five years, the resulting annualized cost ($3,763) would still be dramatically lower than that of haemodialysis. We also assumed that the variable costs from a haemodialysis study conducted from November 2012 to April 2013 [4] still apply. If current costs were higher, the cost advantage of transplantation would be even more favorable. Even if electricity costs had doubled, the cost per patient would have increased by only 2%. Data on costs and outcomes of haemodialysis and transplantation come almost exclusively from only one facility (YGH). However, that facility is a referral hospital, receiving patients from all over the country and beyond. Yaoundé General Hospital (YGH) does the bulk of hemodialysis amongst the 14 sites in Cameroon and the analysis and reporting of the haemodialysis cohorts followed established STROBE guidelines. Furthermore, some of the haemodialysis data used in this paper were drawn from previous studies conducted in other centers in Cameroon. Finally, although missing data due to attrition represent only around 6% in each haemodialysis cohort (6/106 for 2012 and 7/118 for 2017), any data gap creates the potential for bias.

## Conclusion

For CKD, transplantation has lower annual costs and a better prognosis than haemodialysis, making it the preferred option. To address resource constraints, Cameroonian officials may wish to prioritize for publicly funded dialysis those candidates expected to need only short-term dialysis (e.g. have only acute kidney failure or a high likelihood of receiving a transplant soon) and strengthen palliative care for patients with terminal conditions. To maximize the benefits of kidney transplantation, Cameroon may wish to prioritize those with the best prognosis based on close tissue match, young age, and freedom from co-morbidities. To allow even poorer patients access to lifesaving treatment, Cameroon may wish to charge families for dialysis and transplantation on a sliding scale that lowers fees for patients from poorer families. A household visit can assess household resources (e.g. house and roof materials, size, and major possessions such as a car or motorcycle).

### What is known about this topic

- *Previous research found that variable costs for haemodialysis averaged $13,581 per patient per year;*
- *Living donor kidney transplantation began in Cameroon in 2021*.

### What this study adds

- *In Cameroon, the overall medical cost per patient per year was $17,681 for haemodialysis and $3,022 for transplantation (assuming the use of generic drugs);*
- *The cost-effectiveness analysis revealed that compared to no treatment, haemodialysis added 2.59 discounted years at an incremental cost of $19,455 per year alive, while transplantation added 9.75 discounted years at a cost of $3,110 per year alive, in Cameroon;*
- *Kidney transplantation economically dominates haemodialysis by adding 7.16 expected years alive while saving $21,678 per patient in lifetime medical costs*.

## Annexes

**Annex 1**: annual fixed and semi-variable cost per patient of maintenance haemodialysis in Cameroon (PDF-131 KB)

**Annex 2**: annual maintenance cost per patient of haemodialysis in Cameroon (2012 - 2013 study), USD (PDF-94 KB)

**Annex 3**: annual direct medical cost of follow-up post-kidney transplant per patient in Cameroon (PDF-78 KB)

**Annex 4**: follow-up and annualized cost of transplantation per recipient (PDF-85 KB)

**Annex 5**: cost-effectiveness calculations (PDF-92 KB)
